# Test data sets for calibration of stochastic and fractional stochastic volatility models

**DOI:** 10.1016/j.dib.2016.06.016

**Published:** 2016-06-21

**Authors:** Jan Pospíšil, Tomáš Sobotka

**Affiliations:** NTIS – New Technologies for the Information Society, Faculty of Applied Sciences, University of West Bohemia, Czech Republic

**Keywords:** Fractional stochastic volatility model, Heston model, Option pricing, Calibration data, Out-of-sample error

## Abstract

Data for calibration and out-of-sample error testing of option pricing models are provided alongside data obtained from optimization procedures in "On calibration of stochastic and fractional stochastic volatility models" [1]. Firstly we describe testing data sets, further calibration data obtained from combined optimizers is visually depicted – interactive 3d bar plots are provided. The data is suitable for a further comparison of other optimization routines and also to benchmark different pricing models.

**Specifications Table**

**Table:** 

Subject area	*Mathematical Finance*
More specific subject area	*Derivative pricing*
Type of data	*Figures Tables*
How data was acquired	*Descriptive plots of testing data sets, data obtained by calibration trials*
Data format	*Raw*
Experimental factors	*Calibration data unchanged, out-of-sample set selection described in*[1]
Experimental features	*Data from optimization routines were obtained using settings as in*[1].
Data source location	*Germany, Europe.*
Data accessibility	*Data is within this article*

**Value of the data**

• The provided data might help to improve methods described in [1].
• New approaches might be compared to the ones used.
• Methodology of the calibration testing might be of interest to both practitioners and academic researchers in the field.

## 1 Data

**Fig. 1:**
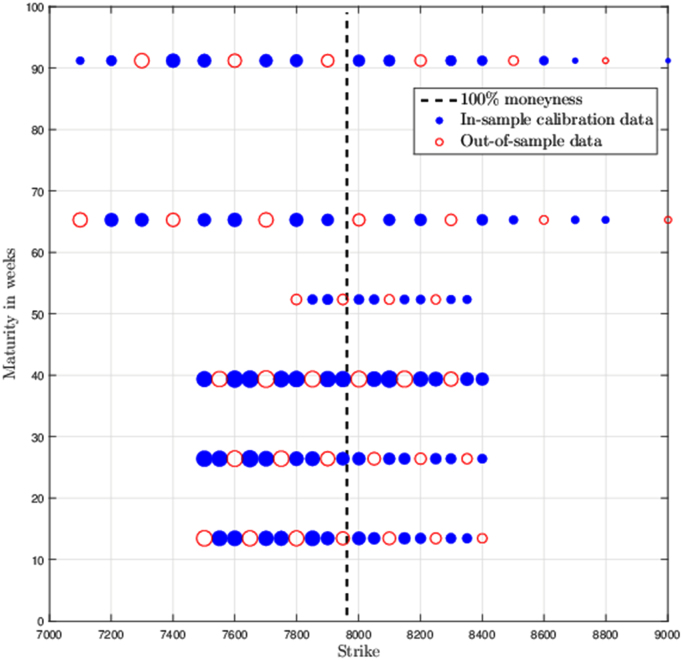
Option price structure in the strike/maturity plane for the secondary data set (19/3/2013) and weights B. Data source: Bloomberg Finance L.P.

**Fig. 2:**
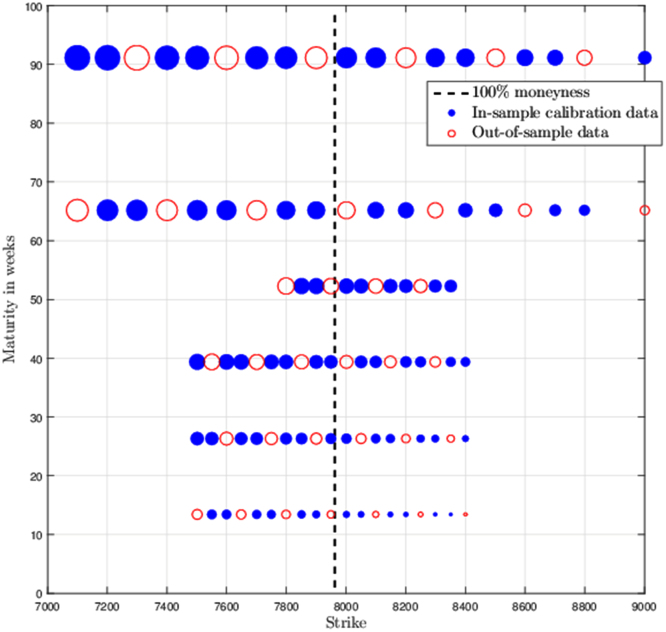
Option price structure in the strike/maturity plane for the secondary data set (19/3/2013) and weights D. Data *source:* Bloomberg Finance L.P.

We describe in detail data sets used for calibration trials in [1]. This includes graphical depictions of option prices in Maturity/Strike plane, see Fig. 1, Fig. 2. A crucial part of any calibration trial is the problem formulation. The following data sets are obtained for a weighted least squares problem formulation, see [1]. Hence, all the upcoming data depends on the weights which we include in separate spreadsheets, see the Supplementary materials.

The data obtained from optimization routines is depicted by figures in Supplementary materials for all sets of weights and both considered models.

## 2 Experimental design, materials and methods

In Fig. 1, Fig. 2 each traded option price is represented by a circle which is centered corresponding to the strike price and maturity of the contract. A circle diameter is proportionate to the weighted option premium. Black dashed line represents 100% moneyness. Figures also depict an in-sample (blue disc) and out-of-sample set (red circle).

Further data were obtained from specific calibration routines, models and problem formulation. The formulation differed only with respect to weights, all employed weight sets (for all contracts) are included in the supplementary spreadsheets. Considered models were the following: the popular Heston stochastic volatility model and the newly introduced approximative fractional volatility model (FSV). Three global optimizers were considered for the calibration task, Genetic Algorithm (GA), Simulated Annealing (SA) and Adaptive Simulated Annealing (ASA). As a local search method a trust region reflective method (LSQ) was used. The most interesting approach in [1], however, was a combination of global methods and the local one. The calibration output data corresponding to these combined methods are visually shown in Supplementary materials.
