# Supplementary figures and images for: Test data sets for calibration of stochastic and fractional stochastic volatility models
Source: Data Brief. 2016 Jun 21;8:628–30. doi: 10.1016/j.dib.2016.06.016 (PMC4936599; doi:10.1016/j.dib.2016.06.016)

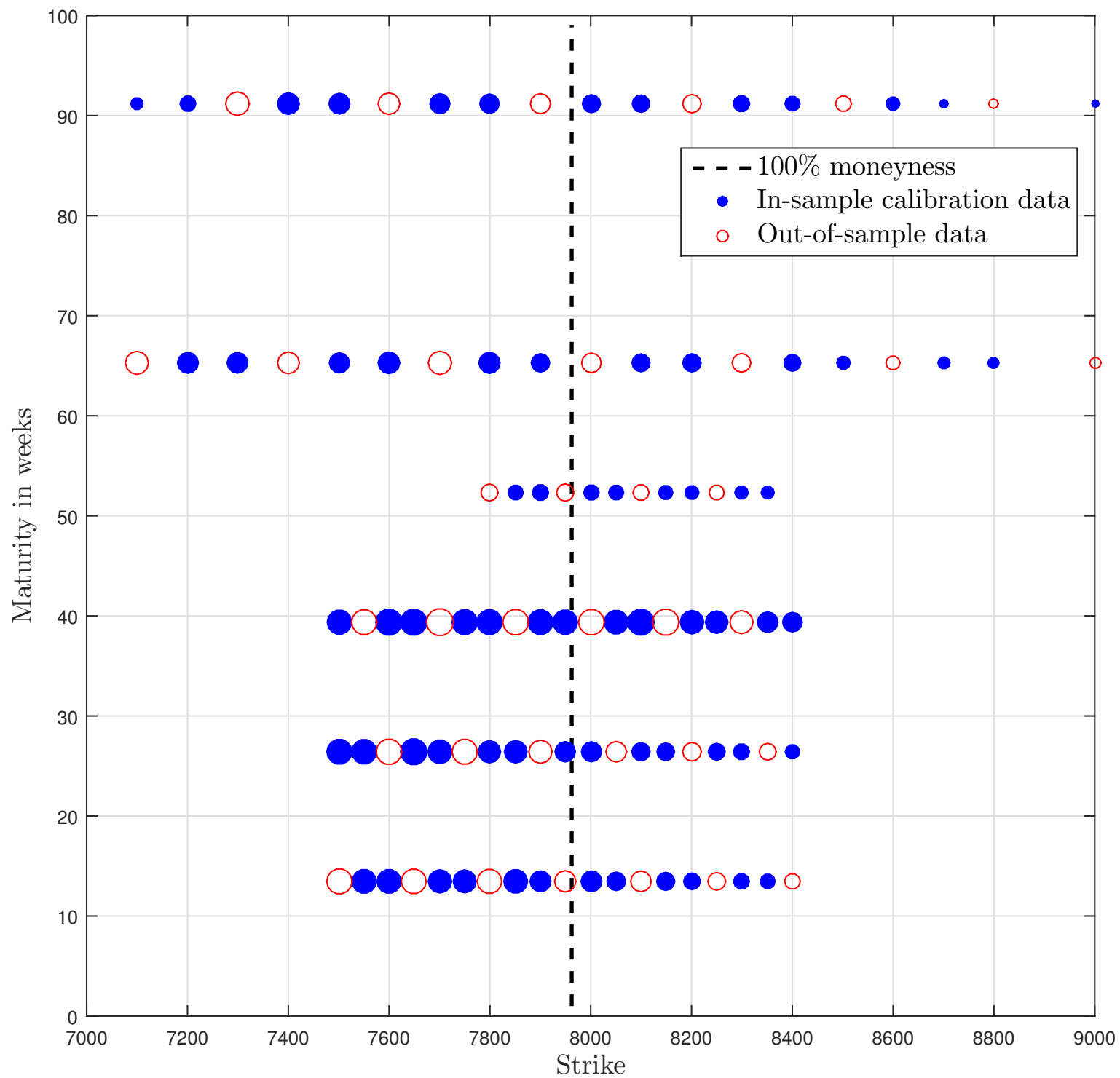

Supplement: Supplementary file 1 — Supplementary material [file mmc1.zip › Figure1.pdf]

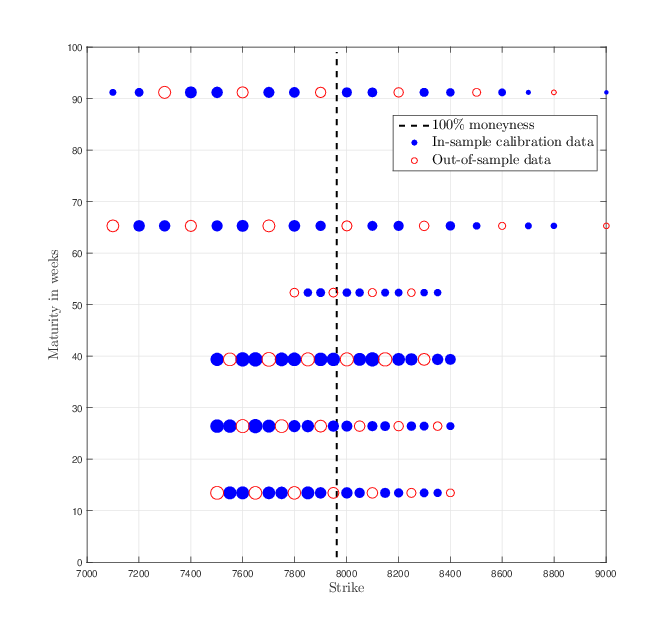

Supplement: Supplementary file 1 — Supplementary material [file mmc1.zip › Figure1.png]

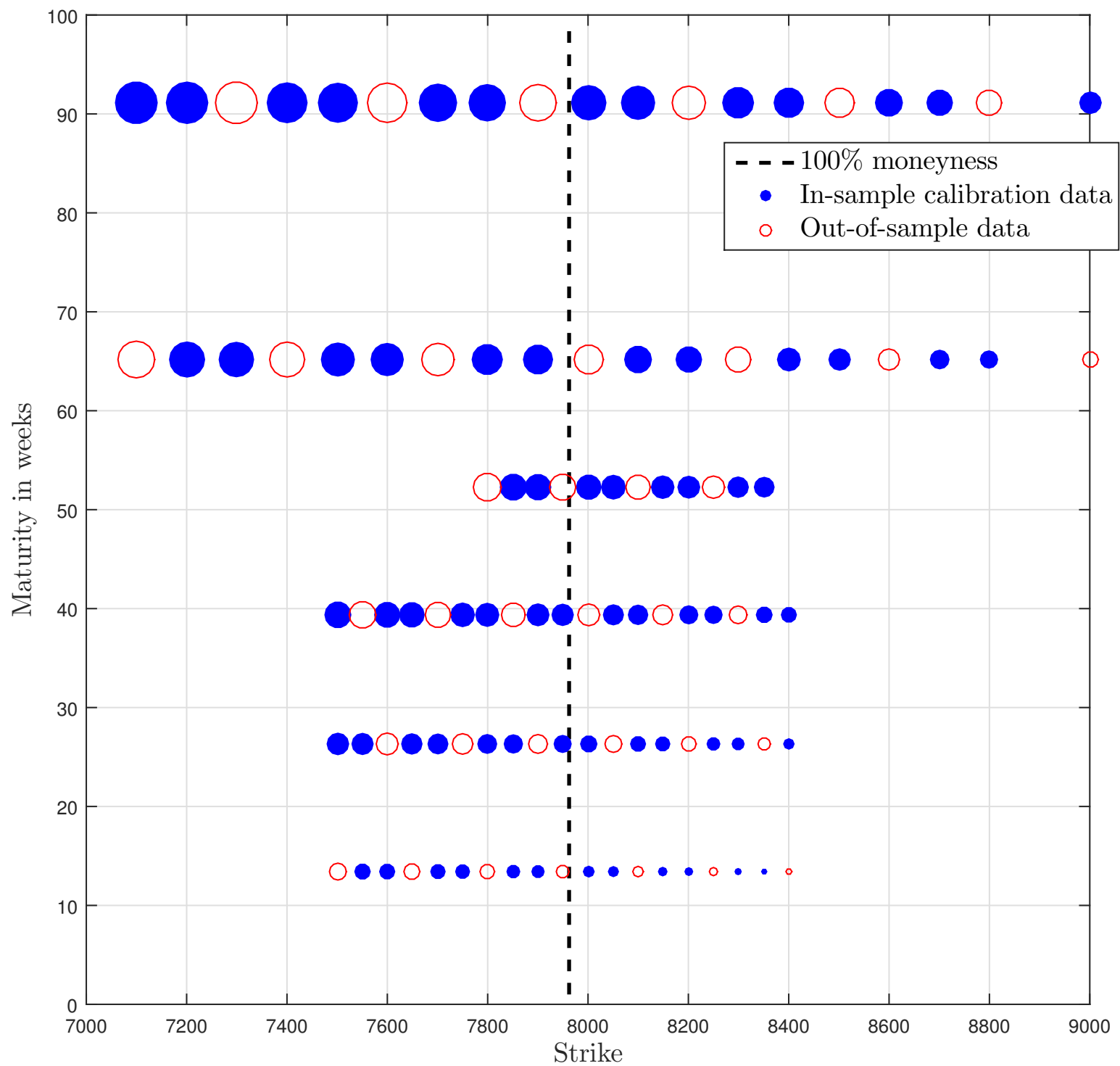

Supplement: Supplementary file 1 — Supplementary material [file mmc1.zip › Figure2.pdf]

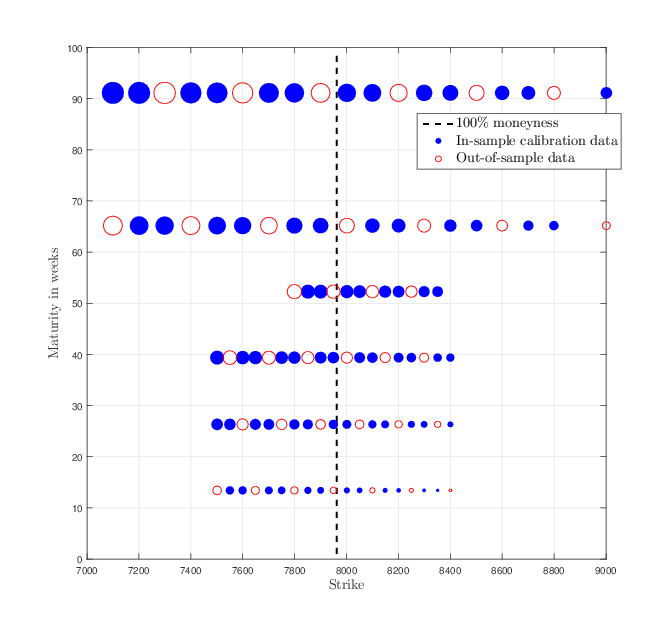

Supplement: Supplementary file 1 — Supplementary material [file mmc1.zip › Figure2.png]
